# Supplementary material for: From sequence to enzyme mechanism using multi-label machine learning
Source: BMC Bioinformatics. 2014 May 19;15:150. doi: 10.1186/1471-2105-15-150 (PMC4229970; doi:10.1186/1471-2105-15-150)
Supplement: Additional file 2 — Java code of ml2db. Additional file ml2db_code.tar.gz contains the Java source code to run the multi-label machine learning experiments and save the results to database. The code’s Javadoc is included. [file 1471-2105-15-150-S2.zip › additional file 2/ml2db/utils/doc/uk/ac/standrews/utils/main/collectionutils/CollectionUtils.html]

CollectionUtils


JavaScript is disabled on your browser.


- Overview
- Package
- Class
- Use
- Tree
- Deprecated
- Index
- Help

- Prev Class
- Next Class

- Frames
- No Frames

- All Classes

- Summary:
- Nested |
- Field |
- Constr |
- Method

- Detail:
- Field |
- Constr |
- Method


uk.ac.standrews.utils.main.collectionutils

## Class CollectionUtils

- java.lang.Object
- - uk.ac.standrews.utils.main.collectionutils.CollectionUtils

- ---

    

  ```
  public class CollectionUtils
  extends java.lang.Object
  ```

  Class

  Version:
  :   1 Jul 2008

  Author:
  :   Luna De Ferrari luna.deferrari-at-ed.ac.uk

- - ### Constructor Summary

    Constructors

    Constructor and Description

    `CollectionUtils()`
  - ### Method Summary

    Methods

    | Modifier and Type | Method and Description |
    | --- | --- |
    | `static int` | `countValues(java.util.Collection collection, java.lang.Object value)` Counts the occurrences of a given value in a collection (using the 'equals' method) |
    | `static java.util.Vector<java.lang.String>` | `fromArrayListToVector(java.util.ArrayList<java.lang.String> list)` |
    | `static java.util.Vector<java.lang.String>` | `fromBigSetToVector(java.util.Set<java.lang.String> set)` Deletes each set entry after writing it to vector, to limit memory use. |
    | `static java.util.Vector<java.lang.String>` | `fromSetToVector(java.util.Set<java.lang.String> set)` |
    | `static java.util.Collection<java.lang.String>` | `removeEmptyStrings(java.util.Collection<java.lang.String> collection)` |
    | `static java.lang.String` | `separatedString(java.util.Vector<java.lang.String> collection, java.lang.String separator)` |

    - ### Methods inherited from class java.lang.Object

      `equals, getClass, hashCode, notify, notifyAll, toString, wait, wait, wait`

- - ### Constructor Detail


    - #### CollectionUtils

      ```
      public CollectionUtils()
      ```
  - ### Method Detail


    - #### countValues

      ```
      public static int countValues(java.util.Collection collection,
                    java.lang.Object value)
      ```

      Counts the occurrences of a given value in a collection (using the
      'equals' method)

      Parameters:
      :   `collection` - a collection
      :   `value` - an object

      Returns:
      :   the count of the object occurrences in the collection


    - #### fromArrayListToVector

      ```
      public static java.util.Vector<java.lang.String> fromArrayListToVector(java.util.ArrayList<java.lang.String> list)
      ```


    - #### fromBigSetToVector

      ```
      public static java.util.Vector<java.lang.String> fromBigSetToVector(java.util.Set<java.lang.String> set)
      ```

      Deletes each set entry after writing it to vector, to limit memory use.

      Parameters:
      :   `a` - big set

      Returns:
      :   a vector of the set elements


    - #### fromSetToVector

      ```
      public static java.util.Vector<java.lang.String> fromSetToVector(java.util.Set<java.lang.String> set)
      ```


    - #### removeEmptyStrings

      ```
      public static java.util.Collection<java.lang.String> removeEmptyStrings(java.util.Collection<java.lang.String> collection)
      ```


    - #### separatedString

      ```
      public static java.lang.String separatedString(java.util.Vector<java.lang.String> collection,
                                     java.lang.String separator)
      ```

      Parameters:
      :   `m_generateNonZeroAttributesNames` -

      Returns:


- Overview
- Package
- Class
- Use
- Tree
- Deprecated
- Index
- Help

- Prev Class
- Next Class

- Frames
- No Frames

- All Classes

- Summary:
- Nested |
- Field |
- Constr |
- Method

- Detail:
- Field |
- Constr |
- Method
